# The Association Between Insurance Status and Cervical Cancer Screening in Community Health Centers: Exploring the Potential of Electronic Health Records for Population-Level Surveillance, 2008–2010

**DOI:** 10.5888/pcd10.130034

**Published:** 2013-10-24

**Authors:** Stuart Cowburn, Matthew J. Carlson, Jodi A. Lapidus, Jennifer E. DeVoe

**Affiliations:** Author Affiliations: Matthew J. Carlson, Department of Sociology, Portland State University, Portland, Oregon; Jodi A. Lapidus, Jennifer E. DeVoe, Oregon Health & Science University, Portland, Oregon.

## Abstract

**Introduction:**

Cervical cancer incidence and mortality rates in the United States have decreased 67% over the past 3 decades, a reduction mainly attributed to widespread use of the Papanicolaou (Pap) test for cervical cancer screening. In the general population, receipt of cervical cancer screening is positively associated with having health insurance. Less is known about the role insurance plays among women seeking care in community health centers, where screening services are available regardless of insurance status. The objective of our study was to assess the association between cervical cancer screening and insurance status in Oregon and California community health centers by using data from electronic health records.

**Methods:**

We used bilevel log-binomial regression models to estimate prevalence ratios and 95% confidence intervals for receipt of a Pap test by insurance status, adjusted for patient-level demographic factors and a clinic-level random effect.

**Results:**

Insurance status was a significant predictor of cervical cancer screening, but the effect varied by race/ethnicity and age. In our study uninsured non-Hispanic white women were less likely to receive a Pap test than were uninsured women of other races. Young, uninsured Hispanic women were more likely to receive a Pap test than were young, fully insured Hispanic women, a finding not previously reported.

**Conclusion:**

Electronic health records enable population-level surveillance in community health centers and can reveal factors influencing use of preventive services. Although community health centers provide cervical cancer screening regardless of insurance status, disparities persist in the association between insurance status and receipt of Pap tests. In our study, after adjusting for demographic factors, being continuously insured throughout the study period improved the likelihood of receiving a Pap test for many women.

## Introduction

Cervical cancer is a significant public health challenge in the United States. Approximately 12,000 women were expected to receive a diagnosis of cervical cancer in 2012, and 4,220 were expected to die of the disease (1). Cervical cancer incidence and mortality rates decreased 67% during the past 3 decades, and most of the decrease is attributed to timely screening and early detection through use of the Papanicolaou (Pap) test. However, early detection efforts are hindered by disparities in cervical cancer screening (2,3). In the general population, 1 factor consistently associated with such disparities is health insurance. Lower rates of cervical cancer screening among uninsured women than among insured women persist across racial, age, and economic groups (4–12).

Community health centers (CHCs) play an important role in addressing disparities in cervical cancer screening. CHCs serve the primary health care needs of over 20 million people in the United States, 38% of whom are uninsured (13). Cervical cancer screening is available to women seeking care in CHCs regardless of their insurance status or ability to pay. As a consequence, disparities in cervical cancer screening among CHC patients related to insurance status should be diminished. However, few studies have investigated the association between insurance status and use of preventive health care services in CHC populations, in part because of a lack of available data. The adoption of electronic health records (EHRs) by some CHCs has now made such research feasible.

We used data from EHRs to assess the association between cervical cancer screening and insurance status in a population of CHC patients. Our objective was to acquire a better understanding of factors affecting use of preventive health care services by CHC patients to inform efforts to reduce health disparities in underserved communities.

## Methods

This cross-sectional study involved secondary analysis of EHR data for a clinic-based population sample. Data were supplied by OCHIN, a nonprofit organization that hosts networked EHRs for CHCs in 13 states. Patient records for women attending 17 clinics located in Oregon and California were retrieved by electronic query from the OCHIN EHR database. These clinics, operated by 5 CHC organizations, were selected because they had both administrative and clinical records available for the entire study period, 2008 through 2010. The study protocol was approved both by the research review committee of OCHIN’s Practice-Based Research Network, which includes CHC representatives, and by the Oregon Health and Science University Institutional Review Board.

### Subject selection

Eligible subjects were women who were aged 24 to 64 years in 2010, who had made 1 or more medical visits at a study clinic during 2010, and, to control for the effects of having a usual source of care (12), at least 1 visit in or before 2008. Patients with a documented history of hysterectomy were excluded. Pregnancy was considered a potential confounder because Pap tests are often administered as part of routine prenatal care and because pregnancy can facilitate access to health insurance. Consequently, subjects who were pregnant during the study period were also excluded.

The outcome of interest was receipt of cervical cancer screening from 2008 through 2010. Receipt of screening was defined by evidence of a completed order for a Pap test in the patients’ EHR. Pap tests performed within 9 months of a prior Pap smear abnormality or related diagnosis of a cervical abnormality were considered diagnostic rather than screening tests, and excluded from the analysis (14). The primary independent variable was health insurance coverage as a percentage of time covered during the period 2008 through 2010. Percentage of time covered was quantified by identifying periods of insurance coverage from the OCHIN database, summing the total number of days with coverage, and dividing by 1,094 days (3 years). To ensure adequate sample sizes for statistical analysis, subjects were classified into only 3 categories: “continuously insured” if they had coverage for 100% of the study period, “continuously uninsured” if they had no coverage during the study period, and “partially insured” if they had coverage for 1% to 99% of the study period. Most insurance records included start and end dates for coverage periods. If the end date was missing, coverage was assumed to have lasted 3 months (15). Insurance coverage that would not pay for cervical cancer screening (eg, workers compensation) was excluded. Periods of coverage totaling less than 7 days were considered administrative errors and excluded.

Covariates included age, race/ethnicity, and household income as a percentage of the federal poverty level (FPL). Selection and categorization of covariates were based on availability of data and informed by previous studies examining use of cervical cancer prevention services (3,5,9,16). The woman’s age, calculated on January 1, 2008, was dichotomized as 21 to 39 years and 40 to 64 years. A combined race/ethnicity variable was generated as follows: women who ever identified as Hispanic or primarily Spanish-speaking were considered Hispanic; among non-Hispanic patients, women who always identified as non-Hispanic white were considered non-Hispanic white; women who met neither of these criteria were classified as non-Hispanic other. Household income as a percentage of FPL was averaged for each participant over the study period, excluding missing records and records with values over 1000%, which were considered erroneous. Categories of 0% to 99% of FPL and 100% or more of FPL were used in analyses.

### Statistical methods

Analysis was restricted to subjects with complete information for all covariates. Descriptive statistics were generated and χ^2^ tests performed to examine differences in distribution of sociodemographic covariates by insurance group. A series of univariable and multivariable bilevel log-binomial regression models was used to estimate unadjusted prevalence ratios (PRs) and adjusted prevalence ratios (APRs) and 95% confidence intervals (CIs) for receipt of cervical cancer screening by insurance status. Log-binomial models were preferred over logistic models, as the latter can strongly overestimate relative risk when the outcome of interest is relatively common (prevalence ≥10%) (17). Patient-level factors were modeled as fixed effects at level 1. A clinic-level variable was entered as a random intercept at level 2 to account for the possible interclass correlation of subjects within clinics (18,19).

Variables associated with the outcome at the *P* < .10 level in univariable models were entered into the multivariable model. Pairwise and 3-way interactions between independent variables were assessed. Backward stepwise selection with an exclusion level of *P* < .05 was used to identify main effects and interaction terms included in the final model. All analyses were conducted in SAS Enterprise Guide version 9.4 (SAS Institute, Inc, Cary, North Carolina).

## Results

Our final study sample included 11,560 women from the base population of 24,382. We excluded a total of 12,822 women, 1,680 who had a history of hysterectomy, 7,943 who had not had an office visit both in 2010 and during or before 2008, 2,683 who were pregnant during the study period, and 516 whose race/ethnicity or FPL data were missing. Six percent (n = 1,217) of Pap tests identified in the EHR were ordered for diagnostic rather than screening purposes and excluded from the analysis.

Within the study sample, 22.9% of women had no insurance coverage from 2008 through 2010, 33.4% had partial coverage, and 44.8% were continuously covered (Table 1). On average, the partially insured were covered for 55% of the study period (range 7%–99%; data not shown). Slightly more than half of subjects (53.9%) were aged 40 years or older. The sample consisted of 46.9% non-Hispanic white women, 38.3% Hispanic women, and 14.8% women classified as non-Hispanic other. Average household income was less than 100% of FPL for 69% of subjects. A total of 63.5% of women received 1 or more Pap tests for cervical cancer screening from 2008 through 2010, but the proportion differed significantly across insurance groups (*P* < .001). Uninsured women were most likely to receive a Pap test (67.6%), followed by the continuously insured (63.9%) and partially insured (60.2%).

**Table 1:** Demographic Characteristics of Women Eligible for Cervical Cancer Screening in Selected Oregon and California OCHIN-Affiliated Community Health Centers (N = 11,560), by Insurance Coverage, 2008–2010

Demographic Characteristic	Total Population, n (%)	No Coverage, n (%)	Partial Coverage, n (%)	Continuous Coverage, n (%)	*P* Value^a^
**Total**	11,560 (100.0)	2,642 (22.9)	3,856 (33.4)	5,062 (43.8)	NA
**Received Pap test**	7,346 (63.5)	1,787 (67.6)	2,322 (60.2)	3,237 (63.9)	<.001
**Age, y**					
21–39	5,324 (46.1)	1,479 (56.0)	1,985 (51.5)	1,860 (36.7)	<.001
40–64	6,236 (53.9)	1,163 (44.0)	1,871 (48.5)	3,202 (63.3)	
**Race/ethnicity**					
Non-Hispanic white	5,426 (46.9)	928 (35.1)	2,246 (58.2)	2,252 (44.5)	<.001
Hispanic	4,424 (38.3)	1,523 (57.6)	880 (22.8)	2,021 (39.9)	
Non-Hispanic other	1,710 (14.8)	191(7.2)	730 (18.9)	789 (15.6)	
**Household income**					
≥ 100% of FPL	3,551 (30.7)	1,286 (48.7)	1,232 (32.0)	1,033 (20.4)	<.001
0–99% of FPL	8,009 (69.3)	1,356 (51.3)	2,624 (68.0)	4,029 (79.6)	

Abbreviations: FPL, federal poverty level.

a *P* values calculated by using χ^2^ test for an association between variable and insurance coverage.

Each independent variable was significantly associated with cervical cancer screening in univariable regression models (Table 2). Hispanics were 1.39 (95% CI, 1.34–1.44) times as likely to receive a Pap test as were non-Hispanic whites; women of other races/ethnicities were 1.16 (95% CI, 1.11–1.22) times as likely as non-Hispanic whites*.* Women aged 40 to 64 years were less likely to receive a Pap test than were women aged 21 to 39 years (PR, 0.93; 95% CI, 0.91–0.96). Cervical cancer screening was more common among the uninsured than among the continuously insured (PR, 1.08; 95% CI, 1.04–1.12). There was no significant difference in receipt of Pap tests between partially insured women and continuously insured women. Women with household incomes less than 100% of the FPL were less likely to be screened than were those with household incomes above 100% FPL (PR, 0.95; 95% CI, 0.92–0.98).

**Table 2:** Prevalence Ratios From Univariable Bilevel Log-Binomial Regression Models^a^ for Cervical Cancer Screening for Women in Selected Oregon and California OCHIN-Affiliated Community Health Centers (N = 11,560), 2008–2010

Demographic Characteristic	N	Women Receiving Pap Test, n (% )	Prevalence Ratio (95% Confidence Interval)^b^
**Total**	11,560	7,346 (63.5)	NA
**Insurance coverage**			
Continuous coverage	5,062	3,237 (63.9)	1 [Reference]
Partial coverage	3,856	2,322 (60.2)	0.98 (0.95–1.01)
No coverage	2,642	1,787 (67.6)	1.08 (1.04–1.12)
**Age, y**			
21–39	5,324	3,531 (66.3)	1 [Reference]
40–64	6,236	3,815 (61.2)	0.93 (0.91–0.96)
**Race/Ethnicity**			
Non-Hispanic white	5,426	2,899 (53.4)	1 [Reference]
Hispanic	4,424	3,337 (75.4)	1.39 (1.34–1.44)
Non-Hispanic other	1,710	1,110 (64.9)	1.16 (1.11–1.22)
**Household income**			
≥ 100% of FPL	3,551	2,335 (65.8)	1 [Reference]
0-99% of FPL	8,009	5,011 (62.6)	0.95 (0.92–0.98)

Abbreviations: FPL, federal poverty level.

a Regression models included patient-level factors as fixed effects at level 1 and a clinic-level random intercept at level 2.

b Prevalence ratios are significant at the α = .05 level if the 95% confidence interval does not contain 1.00.

In addition to main effects, the final multivariable model for estimating prevalence of cervical cancer screening included 3 pairwise interactions: insurance coverage by age (*P* = .013), insurance coverage by race/ethnicity (*P* = .001), and age by race/ethnicity (*P* = .007), plus one 3-way interaction, age by race/ethnicity by insurance coverage (*P* <.0001). Given these interactions, APRs for receipt of a Pap test by insurance status were reported in subgroups defined by race/ethnicity and age (Figure). Among non-Hispanic white women, the uninsured were less likely to receive a Pap test than were the fully insured (APR, 0.80; 95% CI, 0.71–0.91 for ages 21–39 and APR 0.88; 95% CI, 0.79–0.97 for ages 40–64). Younger non-Hispanic white women who were partially insured were also less likely to receive a Pap test than were their peers with continuous insurance (APR, 0.91; 95% CI, 0.84–0.99). Among Hispanic women aged 21 to 39 years, the partially insured were less likely to receive a Pap test than were the continuously insured (APR, 0.91; 95% CI, 0.84–0.99), whereas the uninsured were more likely to receive a Pap test (APR, 1.11; 95% CI, 1.05–1.18) than were the fully insured. Among Hispanic women aged 40 to 64 years, having no insurance lowered likelihood of receiving a Pap test (APR 0.80; 95% CI: 0.75–0.86). There was no significant association between insurance status and cervical cancer screening among women in the non-Hispanic-other category. Household income was an independent predictor of Pap test screening. Women with household income less than 100% of FPL were less likely to be screened than women with income 100% or more of the FPL (APR, 0.95; 95% CI, 0.92–0.97).

**Figure:**
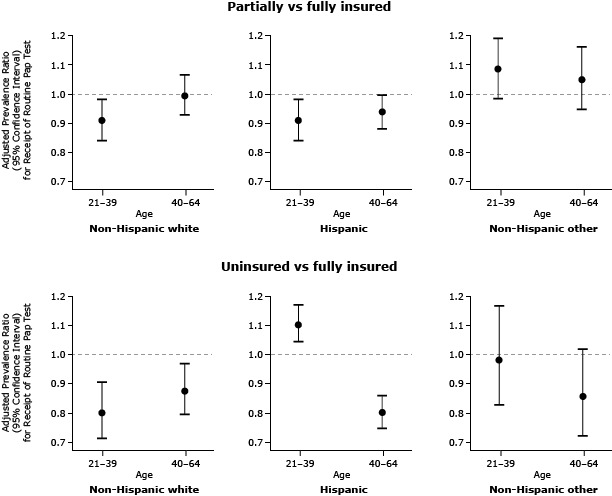
Adjusted prevalence ratios (APRs) and 95% confidence intervals (CIs) for receipt of cervical cancer screening, by insurance status and stratified by race/ethnicity and age, for women in selected Oregon and California OCHIN-affiliated community health centers, from 2008 through 2010 (N = 11,560). Adjusted prevalence ratios are significant at the α = .05 level if the 95% confidence interval does not contain 1.00. **Table:** 

Insurance Comparison and Race/Ethnicity	Age, y	Adjusted Prevalence Ratio (95% Confidence Interval) for Receipt of Papanicolaou Test
**Partially vs fully insured**		
Non-Hispanic white	40–64	1.00 (0.93–1.07)
	21–39	0.91 (0.84–0.99)
Hispanic	40–64	0.94 (0.88–1.00)
	21–39	0.91 (0.84–0.99)
Non-Hispanic other	40–64	1.05 (0.95–1.17)
	21–39	1.09 (0.99–1.20)
**Uninsured vs fully insured**		
Non-Hispanic white	40–64	0.88 (0.79–0.97)
	21–39	0.80 (0.71–0.91)
Hispanic	40–64	0.80 (0.75–0.86)
	21–39	1.11 (1.05–1.18)
Non-Hispanic other	40–64	0.86 (0.72–1.02)
	21–39	0.98 (0.83–1.17)

## Discussion

Our study demonstrates how EHRs enable population-level surveillance among the uninsured and underinsured without resorting to cost- and time-intensive patient surveys. Over 2,600 women who had no health insurance from 2008 through 2010 were included in this study, along with 3,856 women sporadically insured during the same period. The analyses performed here could not have been conducted by using claims data, which misses services received during periods without insurance coverage. Other methods of medical chart abstraction would be impractical given the size of the analytic samples.

Overall, 64% of the women in the study population received a Pap test, a lower proportion than reported in the 2010 National Health Interview Survey (83%) (20), the 2005 Health Information National Trends Survey (90%) (21), and 2010 Behavioral Risk Factor Surveillance System survey results for Oregon (80%) or California (87%) (2). Although the results of our study are not directly comparable with survey data, they add to a growing body of evidence indicating that use of patient self-report data can lead to overestimates of cancer screening (14,22–24). In contrast, screening estimates reported here are higher within equivalent age and race/ethnicity categories than described in the only other known example of a large-scale EHR-based cervical cancer screening study focused exclusively on CHCs (16). Data from 10 Florida CHCs showed that 36% of non-Hispanic white women and 64% of Hispanic women received a routine Pap test from 2005 through 2007 (16). The equivalent proportions reported in our study were 53% of non-Hispanic white women and 75% of Hispanic women. Interestingly, no difference in prevalence of Pap testing was observed between insured and uninsured women in Florida (53% in both groups received a Pap test), whereas being uninsured was a positive predictor of cervical cancer screening in unadjusted estimates reported here. The disparate findings may reflect the use of different inclusion criteria in the 2 studies. For example, the Florida study did not limit subjects to established patients. In addition, clinics participating in the Florida study were in different stages of EHR implementation. Therefore, clinical data were not available for the entire study population (16). Such factors may have resulted in less complete data capture and underreporting of Pap tests in Florida, leading to lower screening estimates than reported here. Insurance status was also defined differently in the 2 studies, potentially contributing to the difference in findings.

In adjusted models, being uninsured lowered the likelihood of receiving a Pap test more for non-Hispanic white women than for Hispanic women and those of other races/ethnicities. Similar results have been reported previously (25), and other studies found lower risks of advanced-stage cervical cancer among uninsured and Medicaid-insured Hispanic women than among similarly insured non-Hispanic white women (26). Such findings have been interpreted to suggest that minority women are more skilled than non-Hispanic white women at accessing free or subsidized screening services offered in safety-net clinics (25,26). Alternatively, the clinics studied here might have cervical cancer screening programs that are particularly effective at reaching underinsured minorities. For example, some or all of the clinics in this study may have participated in the National Breast and Cervical Cancer Early Detection Program (NBCCEDP) from 2008 through 2010, which offers Pap tests to underserved and underinsured women. Although it was not possible to assess the proportion of the study population who were screened through such programs, it is conceivable that initiatives such as the NBCCEDP reduced or even reversed disparities in screening between insured and uninsured or underinsured women, and between women in different age and race/ethnicity groups, that might otherwise have been more evident. Lower rates of Pap test screening among uninsured non-Hispanic white women in this study population may also reflect particular access-to-care barriers specific to this group. For example, non-Hispanic white women in the study include many recent immigrants from Eastern Europe who have diverse linguistic and cultural backgrounds. Linguistic and cultural factors have been previously observed to influence use of preventive health services in primary care settings (27).

The most intriguing finding in this analysis was that uninsured Latinas aged 21 to 39 had a higher likelihood of cervical cancer screening than their peers who were continuously insured throughout this study. While the effect was modest (APR, 1.11; 95% CI, 1.05–1.18), no other examples were found in the literature that document higher Pap test rates for the uninsured than for the fully insured, when controlling for other demographic factors. The result is unlikely to be explained by NBCCEDP participation, because the program is aimed almost exclusively at women aged 40 years or older. It is possible, however, that the study clinics may have concurrently participated in state, county, or CHC-led programs that emphasized cervical cancer screening for younger uninsured Hispanic women, thus contributing to the observed findings. A more detailed analysis of screening rates by clinic may provide useful insight into factors underlying this result. Alternatively, the elevated screening rate among uninsured younger Latinas may be linked to the women’s country of birth. Rodríguez et al (5) found that after adjusting for confounding variables, foreign-born status was positively associated with receiving a Pap test and mammography among Latinas in California. Moreover, foreign-born Latinas were found to have disproportionately lower rates of insurance compared with US-born Latinas and non-Hispanic whites (5). If the uninsured younger Latinas we studied were more likely to be foreign-born than insured Latinas, such a relationship might explain the findings. Additional research on subpopulations within the analytic sample would be required to identify associations between country of birth and screening use. Although beyond the scope of this study, such work could inform public health strategies to improve cervical cancer screening rates among the diverse population of women seen in CHCs.

This study has several limitations. First, data were limited to selected CHCs in Oregon and California, so the results may not be generalizable. Second, receipt of Pap tests was identified via search algorithms with commonly used procedure and diagnostic codes, and a small percentage of services may have been missed if coded differently. The potential for this type of error is probably minor because search algorithms were based on validated scripts developed for federal reporting to the Uniform Data System (28). Automated data extraction from EHRs has also demonstrated good-to-excellent agreement with manually extracted data, with electronic methods having notably larger case capture (29).

Third, no information was available regarding Pap tests received outside the OCHIN CHC network. Consequently, Pap test screening in the study population may be underestimated. The results may also be biased if patients more likely to seek care outside the study clinics were disproportionally distributed among the insurance groups. This could explain some of the differences observed. To minimize the potential for underreporting, the study sample was restricted to established patients. Prior research indicates that women having a usual source of care may be less likely to seek routine cervical and breast cancer screenings elsewhere (6,9,12,30,31). The completeness of OCHIN’s preventive service utilization data has also been validated in other studies (32), and fewer services could be expected to be missing from the OCHIN data among the uninsured because persons without coverage have limited options as to where they can access care (32).

Fourth, because of inconsistencies in data collection at clinic sites, duration of coverage by private insurance may have been over- or underestimated, potentially resulting in misclassification of patients by insurance category. To test this possibility, the data were reanalyzed limiting insured patients to Medicaid and Medicare recipients only. Resulting APRs for receipt of Pap tests by insurance coverage were essentially the same, suggesting that misclassification by insurance status is not a major concern. The use of a single category to define partial insurance may also be considered a limitation, potentially masking dose–response associations between time covered and receipt of cervical cancer screening among partially insured subjects. Although beyond the scope of this study, a subanalysis of partially insured subjects using narrower bands of coverage would allow assessment of such trends.

Fifth, regression models did not explicitly account for organizational differences among the CHCs that could influence receipt of cervical cancer screening, such as provider demographics, clinic participation in prevention programs, and scope of services offered. Finally, this report does not address overuse of Pap tests, a topic of emerging interest as the US health care system struggles to contain costs. EHR data have been successfully used to identify Pap tests performed sooner than recommended for women at low risk of cervical cancer (33). With modification to include new screening recommendations and revised eligibility criteria, the algorithms used here could be adapted to identify such Pap tests, informing efforts to maximize the use of CHCs’ limited resources for provision of appropriate services.

Despite these limitations, this study highlights the utility of EHRs for population-level surveillance among the uninsured and underinsured, demonstrating how EHRs can be leveraged to reveal patterns of preventive health care service use in CHC patients. The results indicate that in this population insurance-related disparities in receipt of cervical cancer screening persist even though Pap tests are available regardless of insurance status. After adjusting for patient demographics, having continuous insurance improved the likelihood of cervical cancer screening for many women studied here. Additional research is warranted to further understand the role of insurance in preventive service uptake among CHC patients.
